# Understanding how the human brain tracks emitted speech sounds to execute fluent speech production

**DOI:** 10.1371/journal.pbio.3001533

**Published:** 2022-02-04

**Authors:** Akiko Callan, Daniel E. Callan

**Affiliations:** 1 Center for Information and Neural Networks, National Institute of Information and Communications Technology, Osaka, Japan; 2 Neural Information Analysis Laboratories, Advanced Telecommunications Research Institute International, Kyoto, Japan

## Abstract

Auditory feedback of one’s own speech is used to monitor and adaptively control fluent speech production. A new study in *PLOS Biology* using electrocorticography (ECoG) in listeners whose speech was artificially delayed identifies regions involved in monitoring speech production.

The main objective of speech articulation is to produce sounds that allow the speaker to effectively communicate with the listener. To accomplish this objective, it is considered that speech production uses both feedforward and feedback control systems. The feedforward system generates articulatory motor commands based on previously learned speech sound maps and predicted auditory states associated with the motor commands. In contrast, the feedback system monitors the difference between the predicted speech sound, based on the motor commands that were made, and the actual speech sounds that occurred. When a mismatch is detected, an error signal is generated, which is used to modify motor commands to compensate for the discrepancy.

The importance of auditory feedback on speech production has been supported by many studies using altered auditory feedback. Delayed auditory feedback (DAF) is one of the most famous methods to alter auditory feedback. In 1950, Lee reported the effects of DAF on speech production [1]. Listening to slightly delayed samples of their own voice through earphones (Fig 1A) caused participants to speak slowly; however, if they attempted to maintain normal speed, artificial stutter characterized by undesired repetition of syllables or fricatives occurred. After this study, many researchers investigated how DAF affects speech production. It is well known that DAF causes nonstutterers to speak disfluently but stutterers to speak more fluently. DAF devices have been developed to help stutterers become more fluent. While the behavioral aspects of DAF effects are becoming clearer, the underlying neural circuitry is still largely unknown.

**Fig 1:**
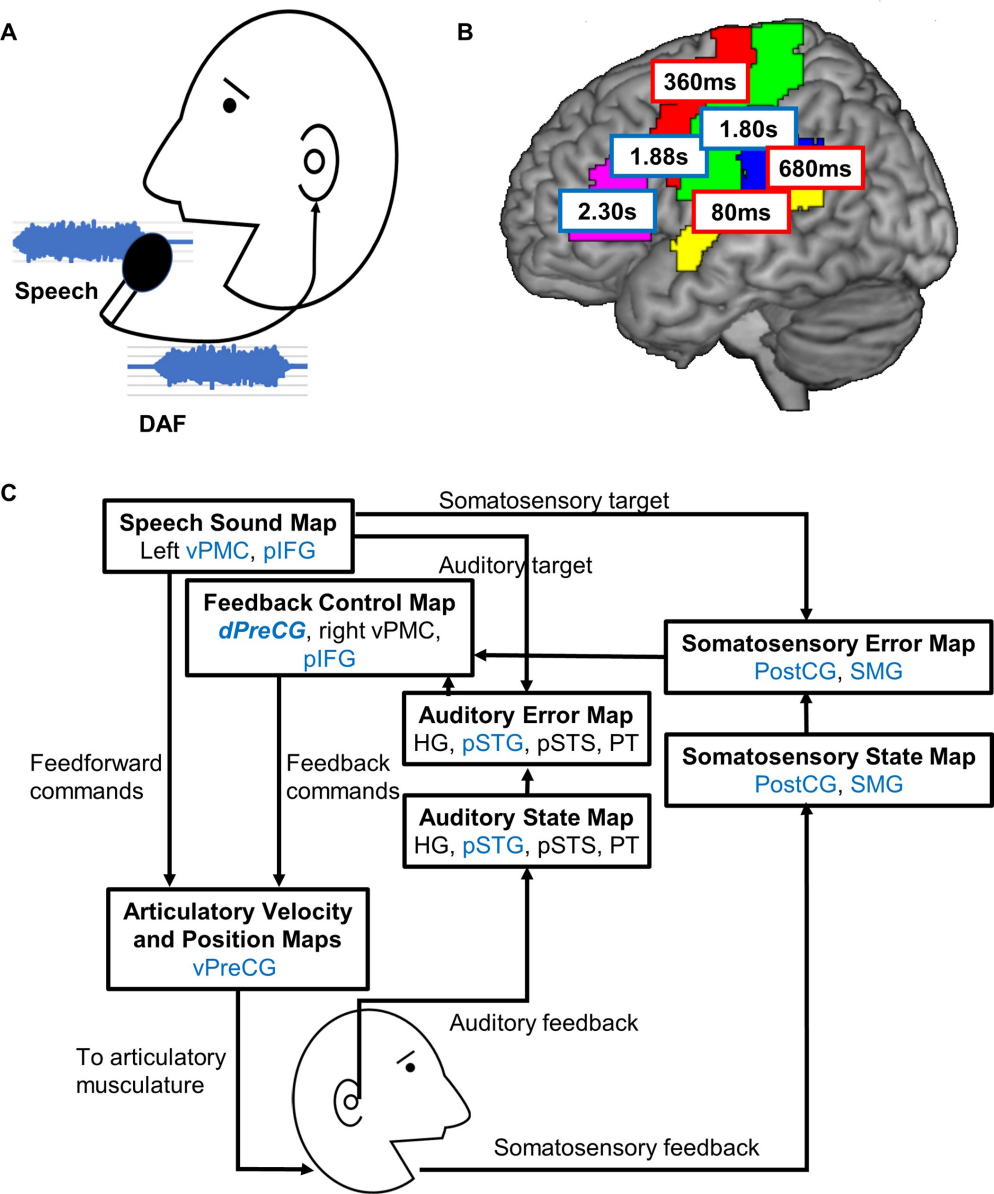
Neural correlates associated with DAF and a cortical model of speech production. **(A)** Experimental setup for the DAF task. **(B)** Divergence onsets when the neural responses to the 4 delay conditions were significantly different at each region. Early onsets are plotted in red rectangles, and late onsets are plotted in blue rectangles. Related gyri are colored in yellow (STG), blue (SMG), green (PostCG), red (PreCG), and pink (IFG). **(C)** A cortical model of speech production based on the DIVA model and Ozker’s study (adapted from Guenther and Vladusich [8]). The dPreCG is a new addition to the feedback control map based on the results of the Ozker and colleagues’ study. The brain regions involved with DAF (STG, SMG, vPreCG, dPreCG, and IFG) from the Ozker and colleagues’ study are shown in blue in the figure. DAF, delayed auditory feedback; dPreCG, dorsal precentral gyrus; HG, Heschl gyrus; IFG, inferior frontal gyrus; pIFG, posterior inferior frontal gyrus; PostCG, postcentral gyrus; PreCG, precentral gyrus; pSTG, posterior superior temporal gyrus; pSTS, posterior superior temporal sulcus; PT, planum temporale; SMG, supramarginal gyrus; STG, superior temporal gyrus; vPMC, ventral premotor cortex; vPreCG, ventral precentral gyrus.

Previous neuroimaging studies using functional magnetic resonance imaging (fMRI) and positron emission tomography (PET) found that signals in the posterior superior temporal gyrus (pSTG: auditory region) are correlated with DAF and are sensitive to the length of delayed feedback [2,3]. Both spatial and temporal characteristics of neural dynamics are important for a better understanding of brain mechanisms. Because the temporal resolution of fMRI and PET is low, obtaining more precise temporal dynamics was needed to account for the DAF effect. Ozker and colleagues provide new insights by using electrocorticography (ECoG) that possesses high temporal resolution [4]. They recorded ECoG signals from neurosurgical patients. The participants heard their own voices with one of 4 different delay types (no delay, 50, 100, or 200 ms), while they were reading visually presented stimuli out loud. A total of 10 different 3-syllable words and 6 different 8-word sentences were used as stimuli.

The behavioral results confirmed that articulation duration increased with delayed feedback and that the elongation effect was greater when people spoke sentences rather than isolated words. By using an unsupervised clustering analysis on neural responses, the authors found 2 response patterns. In the first pattern that was mainly located in the superior temporal gyrus (STG) indicating auditory function, neural responses started after speech onset, and their amplitude increased significantly with delayed feedback. In the second pattern that was mainly located in the precentral gyrus (PreCG) indicating motor function, neural responses started before speech onset, and their amplitude was only affected by delayed feedback during reading sentences.

Further investigation of the DAF effect was performed using regions of interest analysis at STG, supramarginal gyrus (SMG), ventral precentral gyrus (vPreCG), dorsal precentral gyrus (dPreCG), postcentral gyrus (PostCG), and inferior frontal gyrus (IFG). STG, SMG, and PostCG are in sensory; vPreCG and dPreCG are in motor; and IFG is in frontal areas. All regions showed larger sensitivity to DAF during sentence reading, but vPreCG, dPreCG, and PostCG failed to show significant differences during word reading. In order to reveal how response enhancement to DAF changed across time, divergence onsets when the neural response to the 4 delay conditions were significantly different were analyzed using the sentence reading conditions. Results indicated 2 distinct time frames: early onsets in STG (80 ms), dPreCG (360 ms), and SMG (680 ms) and late onsets in PostCG (1.80 s), vPreCG (1.88 s), and IFG (2.3 s) (Fig 1B). However, because articulation duration increased significantly with delay, DAF leads to not only auditory error processing but also longer motor processing. The early and late onsets may indicate auditory and motor natures of the effects, respectively. After controlling for articulation duration, the response enhancement by DAF disappeared in PostCG and vPreCG, indicating that their responses were motor in nature. Based on these results, the authors propose that STG, dPreCG, and SMG are involved in auditory feedback processing and that dPreCG is a critical region for maintaining speech fluency when dynamic auditory feedback processing is required to produce longer utterances.

Guenther and colleagues have proposed a comprehensive neural network model of speech production based on their computational model, called DIVA, and neuroimaging studies [5–9]. According to the model, STG monitors the auditory error, and SMG monitors the somatosensory error. In this model, the feedback controller that transforms auditory errors into corrective motor commands is in the right ventral premotor cortex. This right lateralized auditory feedback control processing was supported by many imaging studies, but those studies only investigated spectral perturbations [10]. If the auditory feedback control system only resides in the right hemisphere, the control system for DAF would not have been identified in Ozker’s study. In this study, brain activity data were recorded through electrodes in 15 patients (2 right, 9 left, and 4 bilateral hemisphere coverage). It is interesting to point out that an fMRI study that investigated both spectral (shifting characteristic frequency) and temporal (elongation of the phoneme) perturbations found involvement of the right vPreCG in spectral feedback control and involvement of the left vPreCG in temporal feedback control [10]. Ozker’s study advances our knowledge about neural correlates associated with temporal feedback control. The study suggests that the auditory feedback controller is located not only in ventral parts but also in dorsal parts of PreCG. Specifically, their results implicate the dPreCG as a critical region for initiating longer speech production under DAF. By applying Ozker’s results to DIVA, we made a modified version of the model (Fig 1C). These findings are an important step in bringing us closer to better understanding the underlying brain mechanisms involved in speech motor control that may lead to advances in treatment for speech disorders.

## Abbreviations

DAF: delayed auditory feedback
dPreCG: dorsal precentral gyrus
ECoG: electrocorticography
fMRI: functional magnetic resonance imaging
IFG: inferior frontal gyrus
PET: positron emission tomography
PostCG: postcentral gyrus
PreCG: precentral gyrus
pSTG: posterior superior temporal gyrus
SMG: supramarginal gyrus
STG: superior temporal gyrus
vPreCG: ventral precentral gyrus
